# OIP5-AS1/CD147/TRPM7 axis promotes gastric cancer metastasis by regulating apoptosis related PI3K-Akt signaling

**DOI:** 10.3389/fonc.2023.1221445

**Published:** 2023-12-13

**Authors:** Jianpeng Chen, Wei Wang, Yujie Zhang, Caixia Wang, Weibo Wang, Aiming Zheng

**Affiliations:** ^1^ Department of Oncology, Shandong Provincial Hospital Affiliated to Shandong First Medical University, Jinan, China; ^2^ Department of Oncology, Dongying Hospital of Traditional Chinese Medicine, Dongying, China; ^3^ Department of Outpatient, Guangzhou University, Guangzhou, China

**Keywords:** gastric cancer, CD147, OIP5-AS1, TRPM7, PI3K-Akt, metastasis

## Abstract

**Background:**

To explore the mechanism of OIP5-AS1/CD147/TRPM7 axis to gastric cancer (GC) metastasis.

**Methods:**

Bioinformatic analysis was performed to pick up the candidate genes associated with regulation GC metastasis. Using GC cell lines, AGS and MKN-45 as research objects, identify the effect of candidate genes on GC metastasis, judge cell proliferation status by MTT assay and cell clone number, and detect cell migration by Transwell and Wound-healing assay. The molecular mechanism of CD147/OIP5/TRPM7 axis regulating GC metastasis was further explored by RNA sequencing. The key signaling pathways were subsequently verified by flow cytometry and WB.

**Results:**

Bioinformatic analysis suggested OIP5-AS1/CD147/TRPM7 axis may be involving in GC metastasis. The RNA interference experiment proved that after gene interference, the proliferation ability of GC cells decreased significantly (*P*<0.05), which was manifested in the reduction of the number of cell clones. In addition, the migration ability of GC cells was also affected, which was based on the results of Wound Healing (*P*<0.05). CD147, OIP5-AS1 and TRPM7 all have harmful effects on GC cells. The relationship between OIP5-AS1 and CD147/TRPM7 was detected by RNA immunoprecipitation. Moreover, the RNA sequencing data indicated that CD147/OIP5-AS1/TRPM7 may coordinately regulate the PI3K-AKT pathway related to GC cell apoptosis, thereby affecting the proliferation and migration of GC cells. After RNA interference, the level of apoptosis increased both in AGS and MKN-45 cells. Meanwhile, the expression of pro-apoptotic proteins Caspase9 and BAX were up-regulated (*P*<0.05). In addition, the expression of PI3K and AKT proteins was reduced (*P*<0.05). The mouse tumorigenesis experiment corroborated the results of the *in vitro* study.

**Conclusion:**

OIP5-AS1/CD147/TRPM7 axis reduces GC cell proliferation by regulating apoptosis associated with PI3K-AKT signaling, further affecting cancer metastasis.

## 1 Introduction

Gastric cancer (GC) is the second leading cause of cancer death worldwide (1). There are many risk factors, including smoking, drinking, male, Helicobacter pylori infection, etc. (2–4). Moreover, genetic factors play an important role in the occurrence and development of GC. Despite the progress of multimodal therapy, the five-year survival rate is still very low. Hence, identification of molecular mechanisms related to gastric cancer progress are very important (5).

Long noncoding RNA (lncRNA) is a kind of RNA with a length of more than 200nt, which regulating gene expression in transcription, translation and post-translation (6, 7). It is widely reported that lncRNA can play a variety of functions by combining with protein, RNA and DNA (8). Opa-interacting protein 5 antisense RNA 1 (*OIP5-AS1*) is a lncRNA first discovered in zebrafish and plays an important role in embryogenesis (9). In cervical cancer cells, OIP5-AS1 prevents HuR gene from binding to its target mRNA to further inhibit cell proliferation. Extracellular matrix metalloproteinase inducer, also known as cluster of differentiation 147 (*CD147*), is participates in many physiological and pathological functions (10). *CD147* increases angiogenesis and EGFR expression by up-regulating VEGF and metalloproteinases, and increases invasion and metastasis by up-regulating MMP. *CD147* can be used as an indicator to estimate GC behavior (11). A study reported that CD147 was correlated with GC (12). Ca^2+^ and Mg^2+^ play an important role in countless cell processes. Among ion channels, transient receptor potential melastatin (TRPM) channels 6 and 7 have similar permeability to the main divalent cations Ca^2+^ and Mg^2+^ (13). Studies have shown that the activation of TRPM7 channel is very important for human head and neck cancer cells (14, 15).

Here, we investigated the effects of *OIP5-AS1*, *CD147* and *TRPM7* to GC by RNA interference. Interestingly, the results indicated that changes in these three genes were able to significantly inhibit the proliferation of GC cells, including AGS and MKN-45 cells. We found that the abnormal expression of *OIP5-AS1/CD147/TRPM7* axis may promote GC metastasis by regulating apoptosis related PI3K-Akt signaling.

## 2 Materials and methods

### 2.1 Cell culture

Gastric cancer cell lines AGS and MNK-45 (Cell Bank of the Type Culture Collection, Chinese Academy of Sciences) were cultured in RPMI 1640 medium supplemented with 10% FBS and 100 U/mL-100 μg/mL penicillin/streptomycin at 37°C.

### 2.2 Experimental design

We used RNA interference and made the following grouping, control group, *siCD147* group, *siTRPM7* group and *siOIP5-AS1* group (n ≥ 3 each group). The above groupings were performed in AGC and MKN-45 cells.

### 2.3 Reverse transcriptase-polymerase chain reaction

Total RNA was extracted through QIAGEN RNeasy mini kit (Germany). AMV reverse transcriptase and random primer (Takara) were used to synthesize total RNA cDNA. General and real-time RT-PCR amplification were carried out by Hotstart Taq polymerase (Takara) and SYBR Premix Ex Taq TM IIt (Takara), respectively. The quantitative PCR reaction was carried out on ABI 7900 system. The reaction was incubated for 5 minutes at 95°C, 40 cycles for 15 s at 95°C and 40 cycles at 60°C. All quantitative PCR reactions were repeated in triplicate. The Ct value of each candidate RNA normalized the expression level of *β-actin* by 2^-ΔΔCt^ method (16).

### 2.4 CCK-8 assay

Cells were seeded into 96-well plates with 2,000 cells per well, and cultured in a carbon dioxide incubator. Cell viability was tested every 24 hrs. Before testing, 10 μL of CCK-8 reagent was added to each well, and cultured at 37°C. Incubate in the box for 1 h, use a microplate reader to detect the OD value with 450 nm excitation light, and draw the proliferation curve.

### 2.5 Colony forming

Briefly, cells from each group were digested and adjusted to a uniform concentration. Then, dilute the cell suspension in gradient multiples, and inoculate each group of cells in a 6-well plate, and place them in a cell incubator for 2-3 weeks. Then, Discard the supernatant, dipped and washed. Finally, 4% paraformaldehyde and crystal violet dye solution were added, respectively.

### 2.6 Transwell assay

Briefly, after incubation in a 24-well plate, drop 500 μL crystal violet staining solution into the hole without chamber. After placed at room temperature for 20 min for dyeing, it was washed with deionized water for 5 times to wash off the background. The cells that migrated to the under-surface of insert membranes were taken photos.

### 2.7 Wound-healing assay

Wound-healing assay was also used for evaluating cell migration. Briefly, after cells attach overnight, create scratches and allow cells to migrate for 24 h. Images were taken under the microscope at 0 and 24 h.

### 2.8 Flow cytometry analysis of apoptosis

After the RNA interference experiment of AGC and MKN-45 cells, the apoptosis was detected. In a short, the cells were performed according to the operating instructions of the Annexin-V fluorescein isothiocyanate/propidium iodide (PI) kit. Subsequently, the FACSCalibur flow cytometer was used for flow cytometry.

### 2.9 Western blot

The expression of protein was determined by Western blot (17). Briefly, the protein was separated on SDS-PAGE and transferred to Hybond membrane. After blocked in 5% milk, the membrane was incubated with primary antibody. Then, it was incubated with IgG coupled with horseradish peroxidase (DAKO). The bands were visualized by ECL-Plus detection reagent (Santa cruz) and analyzed by Image software.

### 2.10 Gene expression analysis in cancer tissue

We downloaded public data deposited in Gene Expression Omnibus (GEO) database [accession number: GSE19826] and The Cancer Genome Atlas Program (TCGA) database, and performed bioinformatics analysis (18).

### 2.11 Identification of different expression genes and function enrichment

DEGs was picked out by using DESeq2 software (19). The differential genes with |log2 Fold change|≥1 and *P*<0.05 were regarded as significant DEGs. Heat maps and volcano maps were used to show the top 200 most differentially expressed genes (DEGs). The volcano map shows up-regulated genes in gastric cancer in red and down-regulated genes in blue.

### 2.12 Protein-protein interaction network analysis

To pick up the key genes may regulate GC metastasis, PPI network analysis was performed (https://string-db.org/). Genes with strong interactions in the network were selected as candidate genes, and the selection threshold is set to *P*<0.05. PPI protein interaction analysis was performed using Metascape database.

### 2.13 Gene correlation analysis

To assess the correlation between key genes, Pearson correlation analysis was used. Scatterplots were performed to show the correlations between genes.

### 2.14 Construction of competing endogenous RNA network

TargetScan software was selected to predict the targeting genes of candidate genes (https://www.targetscan.org/vert_80/). Next, Venn diagram was used to show common target genes among different genes. Then, common target genes were selected to build ceRNA network (20).

### 2.15 RNA immunocoprecipitation

Labeled RNase-free EP tubes, including target antibody group, input group and IgG group, were added with resuspended magnetic bead suspension respectively, and the supernatant was discarded. Added RIP buffer and antibody (anti-CD147: ab108308, anti-TRPM7: ab262698, Abcam) to the EP tube for incubation. Then, 100 μl of cell lysate was added to incubate overnight. Then the magnetic beads were washed with RIP buffer to obtain magnetic bead products, and then RNA was purified. The expression of OIP5-AS1 was detected by qRT-PCR.

### 2.16 Immunohistochemistry

Human gastric cancer tissue was fixed in 4% formaldehyde for 24 h, then dehydrated by gradient alcohol and soaked in wax solution for 24 h after xylene was transparent. Then it was made into 5 μm tissue sections. After xylene dewaxing and 75% alcohol hydration, the tissue sections were placed 5 min a autoclave with sodium citrate solution for 5 min. Endogenous peroxidase blocker was added dropwise and incubated at room temperature for 10 min. Then seal the tissue section with milk. Droped primary antibody (anti-CD147: ab108308, anti-TRPM7: ab262698, Abcam) into tissue sections and incubated at 4°C overnight, then incubated with B and C solutions for 15 min respectively. Then, the chromogenic solution was added dropwise and incubated at room temperature for 10 min, then stained with hematoxylin and weathered with hydrochloric acid and alcohol. Finally, the sections was dehydrated by gradient alcohol and sealed, and the results were observed under the microscope.

### 2.17 Tumor metastasis assay

Established a xenograft model by nude mice. The 30 nude mice were sourced from Beijing Huafukang Biotechnology Co., LTD. In the transfer test, nude mice (n = 10/group) were randomly given AGS target cells (2 × 10^6^ cells, 100µL PBS) through the tail vein. The nude mice were divided into three groups, named AGS-control, AGS-TRPM7-interference, AGS-OIP5-AS1-interference, and AGS-CD147-interference. Firstly, lung tumor formation in nude mice was observed, general clinical photos were taken, and one classic photo of lung tissue was selected for each group. Western Blotting was used to detect the contents of pI3K, p-pI3k, AKT and p-AKT in gastric tissue and lung tissue. Western Blotting was used to detect the expression of Bcl2, Bax, cleaved caspase3 and cleaved caspase 9 in gastric tissues.

### 2.18 Statistical analysis

The data was analyzed by SPSS 10.0 software and expressed as mean standard deviation. Mann-Whitney U was used to distinguish the mean value. *P*<0.05 indicated the difference was statistically significant.

## 3 Results

### 3.1 Identification of key genes affecting GC metastasis

**Figure 1:**
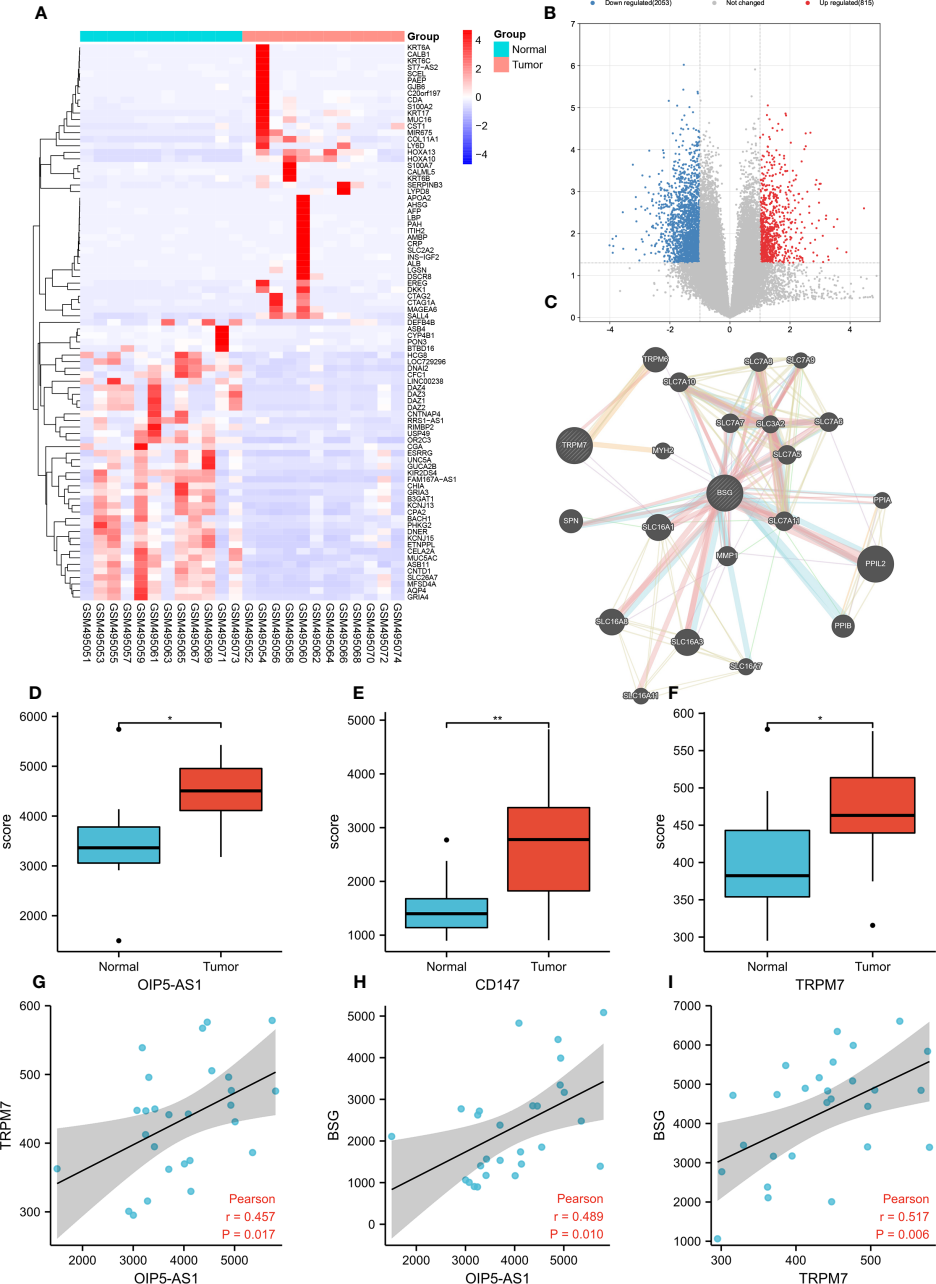
Bioinformatic analysis screen out hub genes in gastric cancer. **(A)** Heatmap showing the top 200 most differentially expressed genes (DEGs); **(B)** The volcano map shows DEGs, with red representing genes up-regulated in gastric cancer and blue representing genes down-regulated in gastric cancer; **(C)** Co-expression network analysis; **(D-F)** Comparison of *OIP-AS1, CD147* and *TRPM7* expression levels; **(G-I)** Correlation analysis between *OIP5-AS1, CD147* and *TRPM7*. * means P<0.05,** means P<0.01.

To explore the DEGs in GC, we conducted bioinformatic analysis based on the GC data of GEO database (GSE19826). Heatmap and volcano map suggested (**Figures 1A, B**) that 2,866 genes were obviously up-regulated in GC, 1,641 genes were significantly down-regulated. Among them, *OIP5-AS1* (**Figure 1D**), *CD147* (**Figure 1E**) and *TRPM7* (**Figure 1F**) were evidently up-regulated in GC tissues. According to PPI network analysis, there was protein interaction between CD147 and TRPM7 (**Figure 1C**). More interestingly, the correlation analysis indicated that there was a positive correlation between *OIP5-AS1* and *TRPM7* (r=0.457, *P*=0.017, **Figure 1G**), and a positive correlation between *OIP5-AS1* and *CD147* (r=0.489, *P*=0.010, **Figure 1H**). *TRPM7* was also positively associated with *CD147* (r=0.517, *P*=0.006, **Figure 1I**). These three genes were positively correlated with each other.

### 3.2 The effects of *OIP5-AS1* on GC

**Figure 2:**
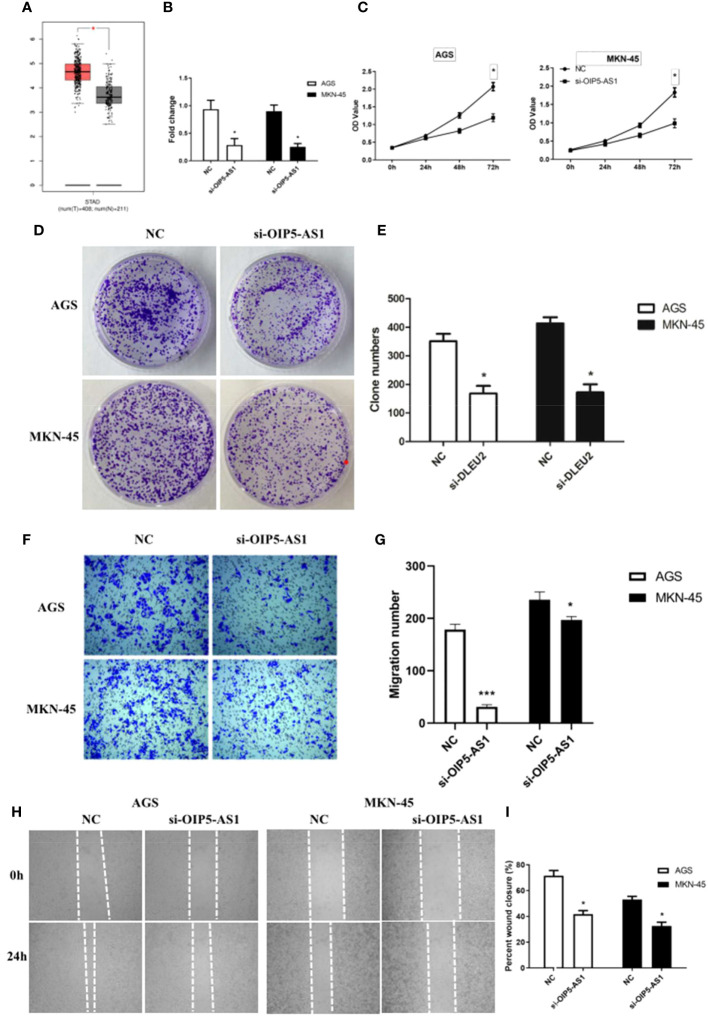
The effects of abnormal expression of *OIP5-AS1* on gastric cancer (GC). **(A)** Box plot showing the expression characteristics of *OIP5-AS1* in normal and cancer tissues; **(B)** *OIP5-AS1* expression level detecting by RT-PCR after RNA interference in GC cell lines, including AGS and MKN-45 cells; **(C)** Cell proliferation curves were examined by CCK-8 assay; **(D)** Detection of cell clone formation by crystal violet staining; **(E)** The bar plot showing the number of cell clones; **(F, G)** The Cell migration; **(H, I)** Wound-healing assay. n ≥ 3 per group, compare to NC, * means *P*<0.05.

*OIP5-AS1* belongs to long noncoding RNA, and it may participate to regulating cancer metastasis because the expression level of *OIP5-AS1* was higher in GC (**Figure 2A**). In order to understand the effect of *OIP5-AS1* on GC, we conducted RNA interference experiments in AGC and MKN-45 cells. Compared to control, the level of *OIP5-AS1* mRNA in RNA interference group was significantly decreased (*P*<0.05, **Figure 2B**). The rate of cell proliferation indicated after RNA interference of *OIP5-AS1*, the cell proliferation efficiency was significantly reduced (*P*<0.05, **Figures 2C, D**). Observation of cell clone clusters showed that in AGS cells, it was reduced by about 50%; in MKN45 cells, it was reduced by 60% (**Figures 2E, F**). Transwell assay showed that *siOIP5-AS1* was able to significantly inhibit the migration of AGS and MKN-45 cells (*P*<0.05, **Figure 2G**). Moreover, Wound-healing assay also supported the results of Transwell assay (**Figures 2H, I**).

### 3.3 The effects of *CD147* on GC

**Figure 3:**
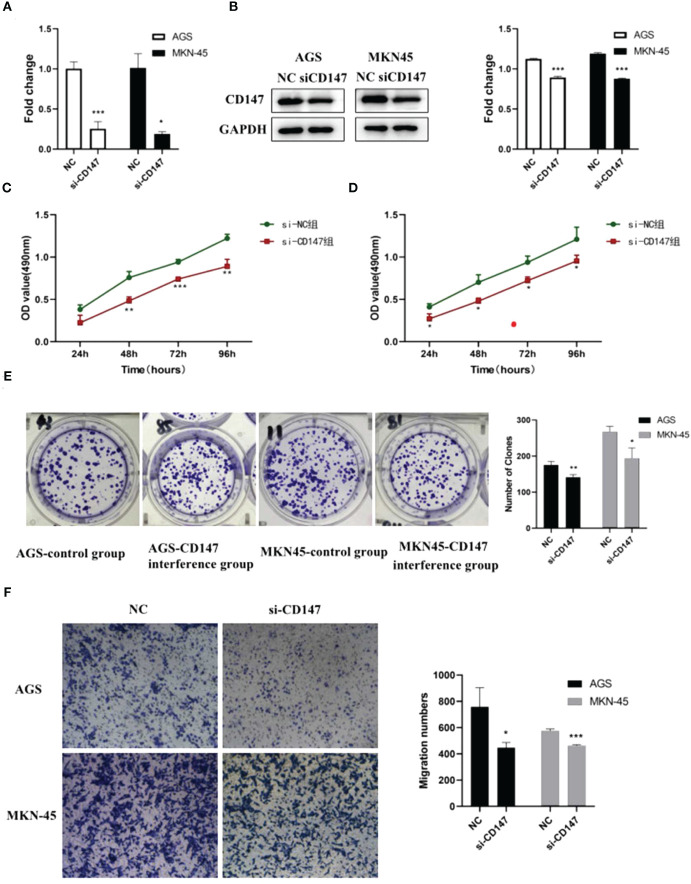
The effects of abnormal expression of *CD147* on gastric cancer (GC). **(A)** *CD147* expression level detecting by RT-PCR after RNA interference in GC cell lines, including AGS and MKN-45 cells; **(B)** CD147 protein expression level detecting by western blot after RNA interference in GC cell lines, including AGS and MKN-45 cells; **(C, D)** Cell proliferation curves were examined by CCK8 assay; **(E)** Clone formation; **(F)** Transwell assay, n ≥ 3 per group, compare to NC, * means *P*<0.05, ** means P<0.01, *** means P<0.001.

Bioinformatic analysis suggested *CD147* may play key role in regulating GC metastasis. However, the effect of *CD147* on GC is still unclear. To address this, RNA interference of *CD147* was performed in two types of GCs, including AGC and MKN-45 cells. Compared to control group, the level of *CD147* mRNA in RNA interference group was significantly decreased, both in AGC and MKN-45 cells (*P*<0.05, **Figure 3A**). As expected, the results of western blot were similar to RT-PCR (**Figure 3B**). The rate of cell proliferation was tested by MTT assay, and it indicated after RNA interference of *CD147*, the cell proliferation efficiency was significantly reduced, both in AGC and MKN-45 (*P*<0.05, **Figures 3C, D**). Observation of cell clone clusters showed that in AGS cells, it was reduced by about 50%; in MKN45 cells, it was reduced by 30% (**Figure 3E**). Transwell assay showed that *siCD147* was able to significantly inhibit the migration of AGS and MKN-45 cells (*P*<0.05, **Figure 3F**).

### 3.4 The effects of *TRPM7* on GC

**Figure 4:**
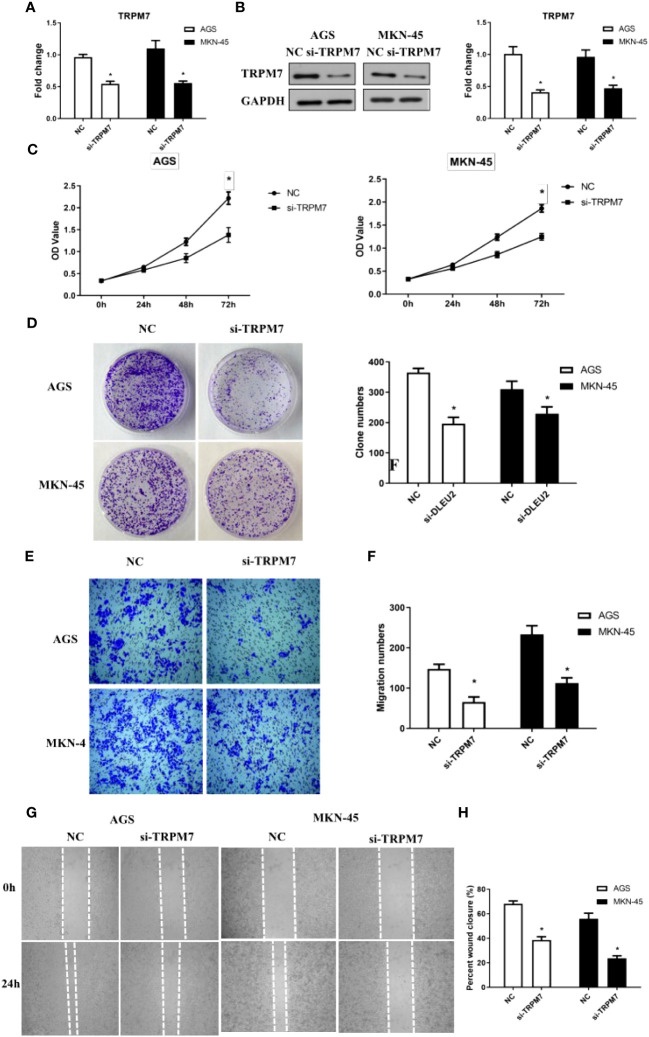
The effects of abnormal expression of *TRPM7* on gastric cancer (GC). **(A)** *TRPM7* expression level detecting by RT-PCR after RNA interference in GC cell lines, including AGS and MKN-45 cells; **(B)** TRPM7 protein expression level detecting by western blot after RNA interference in GC cell lines, including AGS and MKN-45 cells; **(C)** Cell proliferation curves were examined by CCK8 assay; **(D)** Clone formation; **(E, F)** Migration assay; **(G, H)** Wound-healing assay. n ≥ 3 per group, compare to NC, * means *P*<0.05.

To investigate the effect of *TRPM7* on GC, RNA interference experiments were performed. Compared to control, the level of *CD147* mRNA in RNA interference group was significantly decreased, both in AGC and MKN-45 cells (*P*<0.05, **Figure 4A**). As expected, the results of western blot were similar to RT-PCR (**Figure 4B**). The rate of cell proliferation indicated after RNA interference of *CD147*, the cell proliferation efficiency was significantly reduced, both in AGC and MKN-45 (*P*<0.05, **Figures 4C, D**). Observation of cell clone clusters showed that in AGS cells, it was reduced by about 50%; in MKN45 cells, it was reduced by 30% (**Figures 4E, F**). Transwell assay showed that *siCD147* was able to significantly inhibit the migration of AGS and MKN-45 cells (*P*<0.05, **Figure 4G**). Moreover, Wound-healing assay also supported the results of Transwell assay (**Figure 4H**).

### 3.5 The competing endogenous RNA network among *OIP5-AS1, CD147* and *TRPM7*

**Figure 5:**
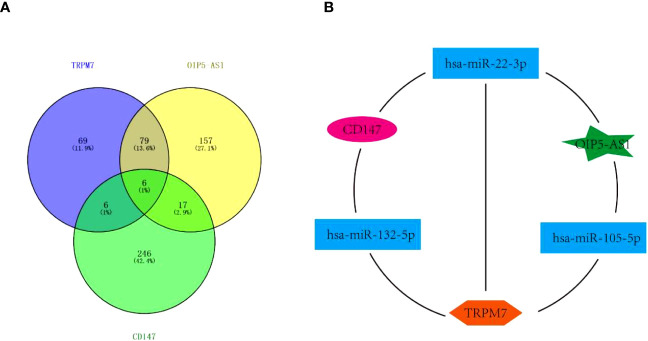
The Competing endogenous RNA (ceRNA) network. **(A)** Venn diagram showing miRNAs targeting *CD147, TRPM7* and *OIP5-AS1*. **(B)** The ceRNA network among *CD147, TRPM7* and *OIP5-AS1.*.

Interestingly, the above data showed that the abnormal expression of *CD147, TRPM7, OIP5-AS1* seems to have the same effect on GC, based on this, we tried to build the ceRNA network relationship between *CD147, TRPM7* and *OIP5-AS1*. TargetScan software was selected to predict the targeting miRNAs of *CD147, TRPM7* and *OIP5-AS1.* The results showed that there were 12 common target genes between *CD147* and *TRPM7*, 23 common target genes between *CD147* and *OIP5-AS1*, and 85 common target genes between *TRPM5* and *OIP5-AS1*. In addition, there are 6 common target genes among them (**Figure 5A**). According to the target gene information, we constructed a ceRNA network (**Figure 5B**).

### 3.6 Disruption of *OIP5-AS1/CD147/TRPM7 axis* triggers GC cell apoptosis

Abnormal expression of *OIP5-AS1/CD147/TRPM7* axis has adverse effects on GC.

**Figure 6:**
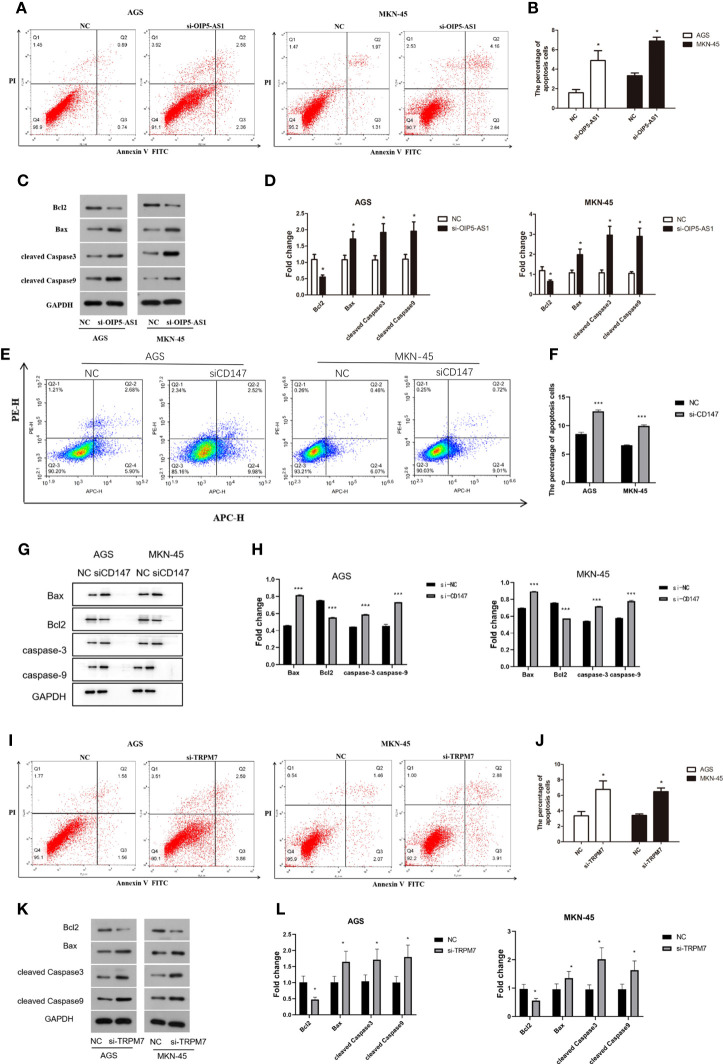
Disruption of *OIP5-AS1/CD147/TRPM7 axis* triggers GC cell apoptosis. **(A)** The apoptosis was detected by flow cytometry after *si-OIP5-AS1.* **(B)** The bar plot showing the apoptosis level both in AGS and MKN-45 cells after *si-OIP5-AS1;* n ≥ 3 per group, compare to control group (NC), * means *P*<0.05; **(C)** Representative images showing the apoptosis related protein; **(D)** The bar plot showing the protein expression level both in AGS and MKN-45 cells after *si-OIP5-AS1;* n ≥ 3 per group, compare to control group (NC), * means *P*<0.05; **(E)** The apoptosis was detected by flow cytometry after *si-CD147.* **(F)** The bar plot showing the apoptosis level both in AGS and MKN-45 cells after *si-CD147;* n ≥ 3 per group, compare to control group (NC), * means *P*<0.05; **(G)** Representative images showing the apoptosis related protein; **(H)** The bar plot showing the protein expression level both in AGS and MKN-45 cells after *si-CD147;* n ≥ 3 per group, compare to control group (NC), * means *P*<0.05; **(I)** The apoptosis was detected by flow cytometry using annexin V-FITC/PI staining after *si-TRPM7;* **(J)** The bar plot showing the apoptosis level both in AGS and MKN-45 cells after *si-TRPM7;* n ≥ 3 per group, compare to control group (NC), * means *P*<0.05; **(K)** Representative images showing the apoptosis related protein; **(L)** The bar plot showing the protein expression level both in AGS and MKN-45 cells after *si-TRPM7;* n ≥ 3 per group, compare to control group (NC), * means *P*<0.05.

The percentage of apoptosis in *OIP5-AS1* interference group was higher than control group, indicating that cell apoptosis was increased (*P*<0.05), both in AGS and MKN-45 cells (**Figures 6A, B**). We next detected the expression level of apoptosis related protein after *OIP5-AS1* interference. The results showed that the Caspase3 and Caspase9 was significantly increased after *OIP5-AS1* interference (*P*<0.05), while the BAX/BCL2 was also increased (**Figures 6C, D**). Subsequently, In AGS and MKN-45 cells, the detection of apoptosis level after *CD147* knockdown showed a significant increase (*P*<0.05) (**Figures 6E, F**), and Western blot experiments showed that pro-apoptosis-related proteins were significantly increased, including Caspase3, Caspase9 and BAX2, and anti-apoptotic protein BCL2 significantly lower expression (*P*<0.05) (**Figures 6G, H**). Moreover, after *TRPM7* was disrupted, it showed a trend consistent with *OIP5-AS1* and *CD147*, that is, the level of apoptosis was significantly increased, which was confirmed by apoptosis flow cytometry and western blot (**Figures 6I–L**).

### 3.7 *OIP5-AS1/CD147/TRPM7* axis promotes apoptosis by impairing PI3K-Akt signaling

**Figure 7:**
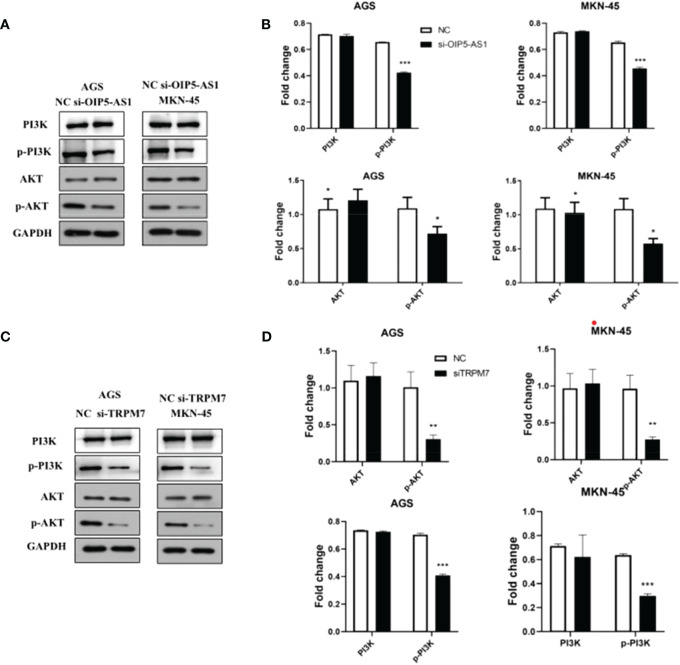
Abnormal expression of OIP5-AS1/CD147/TRPM7 axis disrupts PI3K-AKT signaling pathway. **(A)** Representative images showing the PI3K-AKT signaling pathway related protein after *si-OIP5-AS1*. **(B)** The bar plot showing the protein level both in AGS and MKN-45 cells after *si-OIP5-AS1;* n ≥ 3 per group, compare to control group (NC), * means *P*<0.05. **(C)** Representative images showing the PI3K-AKT signaling pathway related protein *si-TRPM7*. **(D)** The bar plot showing the protein expression level both in AGS and MKN-45 cells after *si-TRPM7;* n ≥ 3 per group, compare to control group (NC), * means *P*<0.05, ** means P<0.01, *** means P<0.001.

Notably, the western blot results showed that the p-AKT protein level was significantly reduced, but there was no significant difference in AKT protein expression after *si-OIP5-AS1* both in AGS and MKN-45 cells. CyclinD1 protein also significantly decreased (**Figures 7A, B**). After *si-TRPM7*, the p-AKT and CyclinD1 protein level was significantly reduced, but there was no significant difference in AKT protein (**Figures 7C, D**).

### 3.8 Verification of OIP5-AS1-CD147-TRPM7 regulatory axis

**Figure 8:**
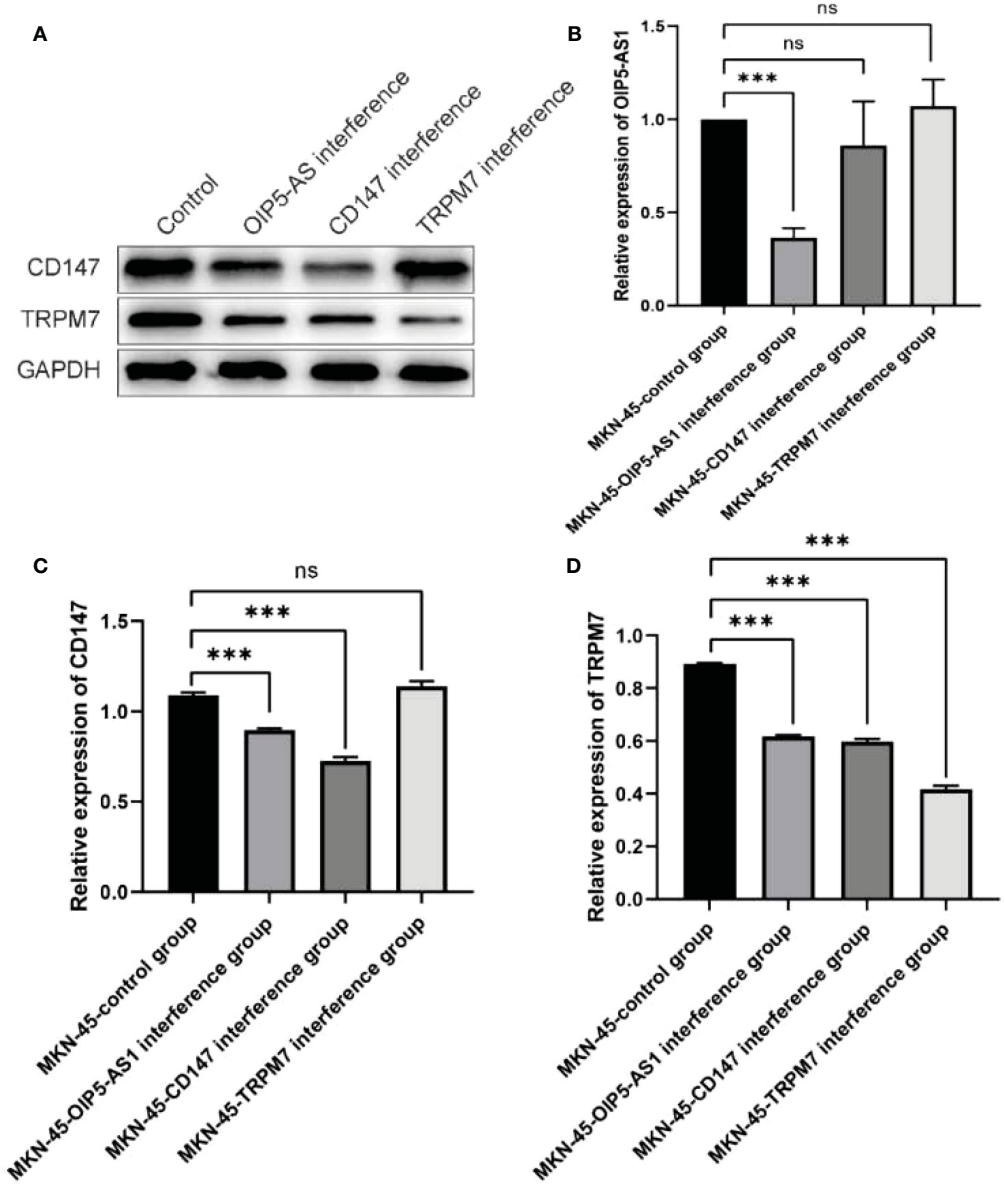
Verification of **OIP5-AS1/CD147/TRPM7** regulatory axis. **(A)** Western blot showed that CD147 and TRPM7 interfered with OIP5-AS1 group, CD147 group and TRPM7 group. **(B)** RT-PCR showed the mRNA expression of *OIP5-AS1* after the same interference with three genes respectively; **(C, D)** Western blot analysis of CD147 and TRPM7. ns means no significance, *** means P<0.001.

In order to further verify the mechanism of *OIP5-AS1/CD147/TRPM7* regulatory axis, we detected the expression changes of each gene after interference of *OIP5-AS1*, *CD147* and *TRPM7* in MKN-45 cell line. The results of western blot indicated that the expression of CD147 was the highest in the normal group and significantly decreased in the OIP5-AS1 group (*P*<0.05). There was no distinct difference in TRPM7 interference group (*P*>0.05, **Figures 8A, C**). Meanwhile, the expression level of TRPM7 was the highest in the normal group and significantly decreased in the other three groups (*P*<0.05, **Figures 8A, D**). Results from RT-PCR showed that *OIP5-AS1* was distinctly down-regulated only in the *OIP5-AS1* interference group (*P*<0.05, **Figure 8B**). The results of this part indicated that the *OIP5-AS1* was located in upstream of the regulatory axis and regulates the expression of *CD147*, which in turn regulates the expression of TRPM7 downstream.

### 3.9 Study on the interaction between OIP5-AS1 and CD147/TRPM7

**Figure 9:**
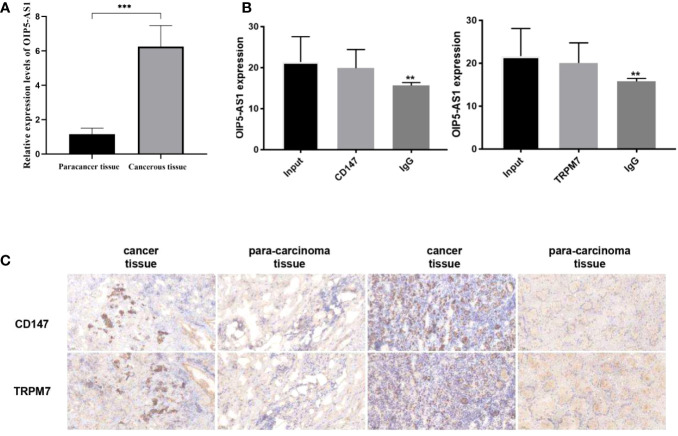
Study on the interaction between OIP5-AS1 and CD147/TRPM7 **(A)** Detected the expression of OIP5-AS1 by qRT-PCR. **(B)** The relationship between OIP5-AS1 and CD147/TRPM7 was detected by RNA immunoprecipitation. **(C)** Detected CD147 and TRPM7 in gastric cancer tissues and adjacent tissues by IHC. n ≥ 3 per group, compare to control group (NC), ** means P <0.01 and *** means P <0.001.

Firstly, we selected human gastric cancer tissues and adjacent tissues, and detected the expression of OIP5-AS1 by qRT-PCR (**Figure 9A**), and detected CD147 and TRPM7 by IHC (**Figure 9C**). The results showed that the positive expression of OIP5-AS1, CD147 and TRPM7 in cancer tissues was higher than that in adjacent tissues. Next, the relationship between OIP5-AS1 and CD147/TRPM7 was detected by RNA immunoprecipitation (**Figure 9B**). OIP5-AS1 and CD147/TRPM7 were combined respectively.

### 3.10 Experiment of tumor formation in nude mice *in vivo*

**Figure 10:**
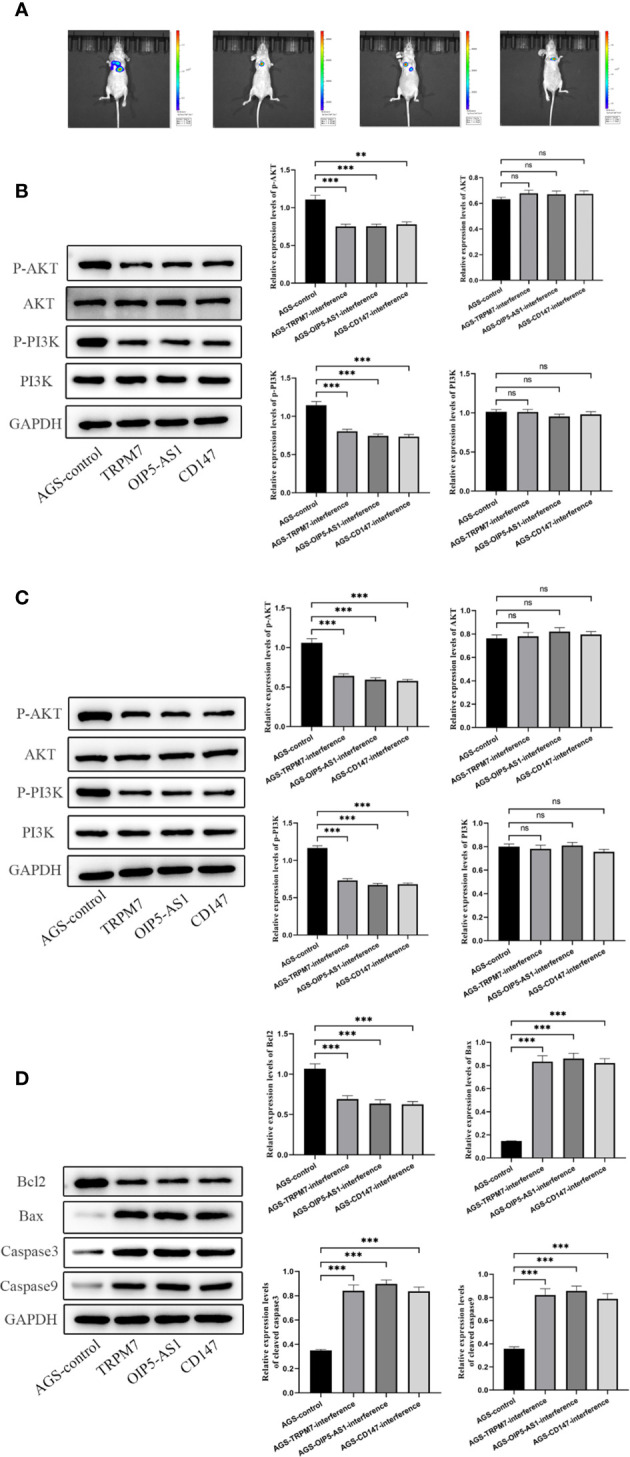
Experiment of tumor formation in nude mice in vivo**(A)** Tumor formation was observed by *in vivo* imaging. **(B)** The contents of pI3K, p-pI3k, AKT and p-AKT in gastric tissue were detected by Western Blotting. **(C)** The contents of pI3K, p-pI3k, AKT and p-AKT in lung tissue were detected by Western Blotting. **(D)** Western Blotting was used to detect the expressions of Bcl-2, Bax, cleavedcaspase-3 and cleaved caspase-9 in gastric tissues. n ≥ 3 per group, compare to control group (NC), ns means no significance, ** means P <0.01 and *** means P <0.001.

Further, we established a mouse tumorigenesis experiment for *in vivo* study. For the observation of lung metastasis and tumorigenesis in nude mice, after three genes were given to mice, the observation of tumor formation in each group showed that lung metastasis was significantly lower than that in control group (**Figure 10A**). Then, the contents of pI3K, p-pI3k, AKT and p-AKT in gastric tissue and lung tissue were detected by Western Blotting. When the three genes were interfered, the contents of p-pI3k and p-AKT decreased significantly compared with the control group. AKT and pI3k contents were the same in all groups (**Figures 10B, C**). Western Blotting was used to detect the expressions of Bcl-2, Bax, cleavedcaspase-3 and cleaved caspase-9 in gastric tissues. After the interference of the three genes, the content of Bcl-2 decreased significantly compared with the control group, and the expressions of Bax, cleavedcaspase-3 and cleavedcaspase-9 increased significantly compared with the control group (**Figure 10D**).

## 4 Discussion

Globally, the incidence of GC ranks fourth among malignant tumors and the second largest incidence in China (21). GC metastasis is one of the important causes of death (22). It is reported that the 5-year survival rate of GC patients is 10-30% due to delayed diagnosis (23). The development and progress of GC is regulated by many factors (24). Because of its high complexity, the current treatment methods cannot achieve satisfactory therapeutic effect (25). Therefore, for the pathogenesis of GC, a deeper understanding is indispensable. Recently a new view shows that cancer is not a kind of hereditary disease, but an ecological disease: a multidimensional space and time of “ecological and evolutionary unity” the pathology of ecosystems (https://www.thno.org/v13p1607.htm). That means genes aren’t everything. Multigene interactions may be one way to shed light on the relationship between cancer progression.

In order to study the pathogenesis of GC, we downloaded the sequencing data located in GEO and TCGA public databases and performed bioinformatics analysis. The results suggested that *OIP5-AS1, CD147* and *TRPM7* were potential key regulatory genes. In addition, correlation analysis showed that these three were positively correlated in pairs, suggesting that there may be a related regulatory axis to regulate GC process. *OIP5-AS1* belongs to lncRNA. LncRNA is involved in the proliferation, growth, migration, invasion and apoptosis of GC (26). At the transcriptional level, lncRNAs regulate mRNA and miRNA expression by altering chromatin modification and mRNA stability, thereby inducing gastric cancer progression (27). Furthermore, lncRNAs regulate GC progression by fully combining with miRNAs to generate ceRNAs or promoting protein stabilization at the post-transcriptional level (28). CD147 is belonging to immunoglobulin (IgG) superfamily (29). It is reported that CD147 is correlated with tumor aggressiveness (30). Chu et al. suggested that the expression of CD147 is associated with GC recurrence and prognosis and is involved in GC metastasis (31). Our results showed that the expression rate of *CD147* was high in two kinds of gastric cancer cells, and the expression of *CD147* mRNA in the CD147 interference group was lower than that in the gastric cancer cell group. TRPM7 is one of the fusion proteins and an enzymatically active kinase domain (32). Activation of TRPM7 channel is very important for human head and neck cancer cells (14, 15). Abnormal expression of TRPM7 has also been reported to be associated with GC prognosis (33).

RNA interference was chosen to explore the role of *OIP5-AS1/CD147/TRPM7* in GC (34). As expected, in two kinds of gastric cancer cells, including AGS and KMN-45 cells, the cell proliferation rate of RNA interference group was lower than that of control group. Regardless of interference with *OIP5-AS1, CD147* or *TRPM7*, the proliferation rate of cells was significantly reduced. The migration rate of GC cells was reduced after RNA interference. All above results indicated that abnormal expression of *OIP5-AS1/CD147/TRPM7* axis affects GC metastasis.

Apoptosis pathway is induced by many signals. One of the mechanisms related to apoptosis is the activation of a series of cytosolic proteases, namely caspase (35). Caspase is synthesized into inactive zymogen, which is processed by autoproteolysis and cleavage of another protein in cells undergoing apoptosis. Functionally, active caspase forms a proteolytic cascade reaction, which can cleave and activate specific substrates. Apoptosis-related proteins include protein inhibitory or promoting factors (36, 37). In two kinds of GC cells, the interference expression of *OIP5-AS1/CD147/TRPM7* could induce the apoptosis of cancer cells by down-regulating the expression of BCL-2 and up-regulating the expression of Caspase3, Caspase9 and BAX. Moreover, the apoptotic proportion of GC cells increased significantly after RNA interference.

Significantly, western blot results showed that the PI3K-AKT signaling pathway changed significantly after the abnormal expression of *OIP5-AS1/CD147/TRPM7*. This was manifested in a significant downregulation of p-AKT protein expression. Alterations in the PI3K-AKT signaling pathway occur frequently in human tumors and are mainly involved in the induction of cell cycle arrest or apoptosis in human tumor cells *in vitro* and *in vivo* (38).

## 5 Conclusion

To sum up, abnormal expression of *OIP5-AS1/CD147/TRPM7* axis promotes GC metastasis by regulating apoptosis related PI3K-Akt signaling.

## Data availability statement

The original contributions presented in the study are included in the article/supplementary material. Further inquiries can be directed to the corresponding authors.

## Author contributions

Conceptualization, Resources, and Funding Acquisition: CW, WBW, and AZ. Methodology, Software, and Validation: JC, WW, and YZ. Formal Analysis: JC. Investigation: WW. Data Curation: WW and YZ. Writing – Original Draft Preparation: JC and WW. Writing – Review & Editing: CW and WBW. Visualization: AZ. Project Administration: WBW and AZ. All authors contributed to the article and approved the submitted version.
